# The London Hospitals

**Published:** 1904-01-02

**Authors:** 


### HOSPITAL ADMINISTRATION.

### CONSTRUCTION AND ECONOMICS.

### THE LONDON HOSPITALS.

BUILDING REQUIREMENTS, RESOURCES, AND ECONOMY.

The discussions which have taken place during the year in regard to the position and requirements of the London hospitals have led to some misapprehensions which we think it may be well to remove. The public and the Press are not unnaturally apprehensive at the financial outlook of the London hospitals. They see ahead appeals for a million and a half of money for rebuilding and reconstruction which must be provided during the next ten years. It is not, however, sufficiently realised that an equally large sum or rather more has been expended by some of these institutions for similar purposes during the last ten years. Further, that when the second £1,500,000 is forthcoming there should be practically an end to these huge appeals for, say, fifty or sixty years. The history of our hospitals shows that great calls have been successfully made for large sums of money for hospital building in the past, and that owing to an awakening of public opinion or the public requirements such appeals have usually come almost together, and have invariably been met. That is to say, hospital rebuilding in the past has been done throughout the country at or about the some period of time. It is not understood, and hence most of the fear that sufficient money may not be supplied by the public, that these special efforts to raise large sums for building are on capital account only, and that when they have been met the provision which results has proved equal to the requirements of the population for decades of years afterwards. It is essential, however, the hospital authorities should realise that when the present building operations are completed, they must organise their finances upon a plan which will enable them to gradually add to their capital year by year, so as to replace the securities which may have been sold at the moment, and to provide them with reserves for building operations in the future.

In order to show that this is a practical proposal, the income and expenditure of the hospitals must be considered in detail. The income and expenditure accounts of the London hospitals to-day prove that for practical purposes when legacies are brought into consideration, as they must and ought to be, owing to the establishment of the King's Hospital Fund and the aid rendered by the Hospital Sunday and Saturday Funds, the public are providing nearly, if not quite, enough money on revenue account, to maintain the London hospitals in efficiency. In other words the appeals on account of capital for building are temporary and special, and they have, fortunately, been delayed until the means for raising the revenue have been organised so as to provide each year the necessary funds for maintaining the hospitals with the maximum of efficiency.

In the year 1901 the total income of the London hospitals was as follows :—

| | General Hospitals. £ | Special Hospitals. £ | Grand Totals. £ |
|---|---|---|---|
| Total Ordinary Income ... | 443,875 | 194,402 | 638,277 |
| Legacies ... ... ... | 134,884 | 131,769 | 266,653 |
| Totals ... ... ... | 578,759 | 326,171 | 904,930 |
| Add Donations for Special Purposes, including Buildings ... ... ... | 140,830 | 56,348 | 197,178 |
| Grand Totals ... ... | 719,589 | 382,519 | 1,102,108 |

The expenditure of the London hospitals in 1901 was :—

| | General Hospitals. £ | Special Hospitals. £ | Grand Totals. £ |
|---|---|---|---|
| *Ordinary* Expenditure ... | 551,564 | 233,721 | 785,285 |
| *Extraordinary* Expenditure | 174,660 | 44,200 | 218,860 |
| Totals ... ... ... | 726,224 | 277,921 | 1,004,145 |

It thus appears that in the year 1901 the London hospitals had an ordinary income, including legacies, of £904,930, and that their ordinary expenditure was £785,285, which left a surplus of about £120,000. The extraordinary expenditure amounted to £218,860, towards which special donations amounting to £197,178 were given, so that on the year the hospitals collectively had a surplus revenue from all sources of nearly £100,000 (£97,962), against which must be charged any special commitments for building purposes for works in progress but not completed and paid for.

The corresponding figures for the year 1902 are not yet available, but the Hospital Sunday Fund returns show that during the twelve months ended December 31st, 1902, the voluntary hospitals and medical charities of London, including convalescent homes and dispensaries, relieved over 1,775,000 patients at a cost of £1,161,133. The ordinary income of these charities amounted to £867,569, and legacies yielded £290,554, making a total revenue of £1,158,123, showing a net deficiency of only £3,010. These figures confirm the view that, providing the public will contribute during the next ten years some million and a half pounds for building and reconstruction purposes, so as to modernise the whole of the London hospitals and bring them up to date, these institutions will be placed once and for all upon a sound financial basis under the voluntary system.

#### The Enforcement of Economy.

The one paramount necessity of the moment is the enforcement of economy in regard to the expenditure of our hospitals in London and elsewhere. The great changes brought about by Lord Lister's discoveries and the introduction of trained nurses and modern methods of treatment have led the authorities into large expenditure, which in the first stages of these developments, owing to a natural desire not to cripple efficiency, has been too prodigal in many directions. The administration of every hospital should be under the direct personal supervision of a resident medical superintendent of the highest capacity, whose duty it should be to exercise control over every item of expenditure in every department, as well as to look after the general administration of the whole hospital. Under such a system it should be possible to reduce the present expenditure by from £10 to £20 per bed. Seeing that there are upwards of 8,500 beds in the London hospitals, the resources of the hospitals might in this way be increased by from £85,000 to £170,000 a year, and the calls upon the public for subscriptions each year would, of course, be correspondingly decreased too.

